# Systematic review of surgical techniques for medial epicondylitis: evaluating the impact of preoperative injections and concomitant ulnar neuritis on postoperative outcomes

**DOI:** 10.1308/rcsann.2025.0005

**Published:** 2025-03-25

**Authors:** A Barakat, G Jha, P Raval, E Abourisha, P Divall, HP Singh, R Pandey

**Affiliations:** University Hospitals of Leicester NHS Trust, UK

**Keywords:** Golf elbow, Medial epicondylitis, Elbow tendinitis, Ulnar neuritis, Flexor pronator

## Abstract

**Introduction:**

Surgical intervention for medial epicondylitis (ME) is indicated when conservative management fails. This review evaluates different surgical techniques for management of ME in terms of patient-reported outcomes (PROs) and complication rates with a focus on the prognostic implications of preoperative injections and concomitant ulnar neuritis on postoperative outcomes.

**Methods:**

Major medical databases were searched for relevant ME studies published between 2000 and September 2023. Case reports, reviews, abstract-only studies and pre-2000 studies were excluded. Two independent reviewers assessed the databases. A best evidence synthesis using Methodological Index for Non-Randomised Studies (MINORS) criteria summarised findings because of study heterogeneity.

**Findings:**

Seventeen surgical studies (442 patients) met the inclusion criteria; most were retrospective (14 studies). MINORS scores ranged from 3 to 14, indicating variable methodological quality. Weighted means showed significant postoperative PRO improvements (*p *> 0.05). The overall complication rate was 3.1%, with percutaneous techniques showing 0% complications vs 6.4% for arthroscopic release and 11.1% for ulnar nerve transposition. Median time to surgery was 6 months of failed nonoperative treatment. Two studies found minimal impact of preoperative ulnar neuritis on outcomes. One of four studies assessing preoperative injections found a significant negative correlation with outcomes.

**Conclusions:**

This review highlights a scarcity of high-quality research on surgical ME management. Nevertheless, surgical treatment for recalcitrant cases shows promising outcomes with low complication rates, particularly for percutaneous techniques. The evidence suggests that neither preoperative injections nor pre-existing ulnar neuritis significantly affects postoperative outcomes in patients undergoing surgery for ME

## Introduction

Medial epicondylitis (ME), colloquially known as ‘golfer’s elbow’, is a musculoskeletal condition characterised by pain and tenderness on the medial side of the elbow. It primarily implicates the flexor carpi radialis and pronator teres, while sparing other components of the common flexor pronator origin.^1^

The treatment for ME is diverse, spanning conservative measures, interventional procedures and surgical interventions. Initially, conservative management including physiotherapy, bracing, activity modification and nonsteroidal anti-inflammatory drugs is typically recommended.^2^ However, if these measures fail to provide relief, transition to alternative approaches becomes necessary. Among these alternatives are various electrophysical interventions such as extracorporeal shock wave therapy, laser therapy or radiotherapy.^3^ Subsequent to unsuccessful conservative methods, initial injections involving steroids, platelet-rich plasma (PRP), or autologous blood are commonly considered as the next step in treatment.^4,5^ Importantly, nonoperative treatment is the hallmark for ME management and should be exhausted before considering any surgical options. Surgery should be viewed as an absolute last resort if all other conservative and interventional approaches have failed to provide adequate symptom relief.

When conservative measures fail to alleviate symptoms, surgical intervention becomes a more appropriate course of action for this condition, including open, arthroscopic or percutaneous release. Several factors influence the decision-making process in selecting among these treatment methods, including the concurrent status of the ulnar nerve, the patient’s hand dominance and the extent of sports participation and occupation. However, consensus remains lacking regarding the necessity of flexor pronator origin (FPO) reattachment after release, the preferred technique for such reattachment (single- or double-row repair) and ideal postoperative rehabilitation protocol (immediate immobilisation vs accelerated rehabilitation). Furthermore, there arises the question of whether pre-existing ulnar neuritis justifies an ulnar nerve procedure (decompression vs transposition), and the optimal approach for diagnosing this pre-existing neuritis, whether solely through clinical means or by additionally confirming it with an electrophysiological study.

ME is much less common than its counterpart, lateral epicondylitis (LE), with ME comprising only 10% of the diagnoses of elbow tendinopathies. Hence it has garnered comparatively limited attention in the published literature on elbow tendinopathies.^6^ Despite the paucity of published literature compared with LE, this condition has a considerable impact on the quality of life.

Although this systematic review aims to provide an overview of surgical techniques for ME, it is important to acknowledge the limitations in the quality of existing studies. The majority of the available studies are retrospective, and the lack of randomised controlled trials (RCTs) poses a challenge in drawing definitive conclusions. This highlights the need for more high-quality research to establish stronger evidence-based guidelines for treatment.

This systematic review aims to evaluate the effectiveness of different surgical techniques for ME, focusing on functional outcomes, complication rates and the impact of preoperative injections and pre-existing ulnar neuritis. The primary research goal is to synthesise the best available evidence to inform clinical decision making. Specifically, we aim to determine the most effective surgical technique and assess whether preoperative factors such as injections and ulnar neuritis influence postoperative outcomes.

## Methods

### Literature database search

This study followed the PRISMA guidelines for systematic review reporting, with the systematic search for this review conducted in September 2023. Computerised literature searches were conducted between January 2000 and September 2023 across various databases, including PubMed/MEDLINE, Biomed Central, BMJ.com, CINAHL, the Cochrane Library, NLM Central Gateway, EMBASE, OVID, AMED, ProQuest (Digital Dissertations), PsycINFO, ScienceDirect and Web of Science. Key search terms such as “Golf Elbow”, “Medial Epicondylitis”, “Medial Tendinopathy” and “Elbow Epicondylitis” were used. In PubMed, Medical Subject Headings terms were utilised where applicable, along with Boolean operators. The full details of the implemented search strategy are available in Appendix 1 (available online). Studies were included if they met the following criteria: published from 2000 onwards, focused on surgical interventions for ME and assessed using the Methodological Index for Non-Randomised Studies (MINORS) criteria. Narrative reviews, case reports and studies predating 2000 were excluded. A secondary search (citation mining) was undertaken, whereby the reference lists of the included articles were reviewed for additional references not initially identified in the primary search.

### Inclusion and exclusion criteria

Interventional studies related to ME from 2000 onwards were included. Exclusions comprised narrative reviews, systematic reviews, case reports, abstract-only publications (presentations) and studies predating 2000. If a study involved both LE and ME but allowed separate stratification and distinction of outcome measures for the ME cohort, it was included. Two authors independently reviewed the articles selected for the selection process. Both titles and abstracts were reviewed to identify potentially relevant articles, and inclusion was based on criteria outlined previously. In cases in which the eligibility of an article was uncertain from the abstract alone, the full-text version was obtained and assessed against the inclusion criteria. All articles meeting the inclusion criteria had their full-text versions retrieved for quality assessment and data extraction.

### Strategy for data synthesis

A quantitative meta-analysis of the studies was not possible owing to the heterogeneity of the study populations, interventions and outcome measures. However, weighted means were calculated for the available outcome measures. The results were also summarised using a best evidence synthesis. The methodological quality of the included studies was evaluated based on two parameters: first, the methodological quality for each study where nonrandomised studies were assessed using MINORS (Table 1); and second, the evidence level according to the hierarchy of evidence established by the Australian National Health and Medical Research Council (Table 2). Two reviewers independently reassessed the data synthesis, and any discrepancies were resolved through a consensus-based approach.

**Table 1:** MINORS criteria for comparative and noncomparative studies

Criteria	Score
Non-comparative studies (total possible score = 14)	
Clearly stated aim	0–2
Inclusion of consecutive patients	0–2
Prospective collection of data	0–2
Endpoints appropriate to the aim	0–2
Unbiased assessment of the study endpoint	0–2
Follow-up period appropriate to the aim	0–2
Loss to follow-up less than 5%	0–2
Additional items for comparative studies (total possible score = 22)	
Comparative analysis between groups	0–2
Baseline equivalence of groups	0–2
Adequate statistical analyses	0–2
Adequate control for confounding	0–2

Scores for each criterion can range from 0 to 2, with higher scores indicating better methodological quality.

**Table 2:** The evidence level according to the hierarchy of evidence established by the Australian National Health and Medical Research Council

Evidence level	Study design
Level I	Systematic review or meta-analysis of RCTs
Level II-1	High-quality RCT
Level II-2	RCT with a significant flaw; e.g. inadequate randomisation, concealed allocation or blinding
Level II-3	Non-randomised controlled cohort/follow-up studies
Level III-1	Evidence obtained from well-designed pseudo-RCTs (alternate allocation or some other method)
Level III-2	Evidence obtained from comparative studies with concurrent controls and allocation not random (cohort studies), case–control studies, or interrupted time series with a control group
Level III-3	Evidence obtained from comparative studies with historical control, two or more single-arm studies, or interrupted time series without a parallel control group
Level IV	Case series (and poor-quality cohort and case–control studies)
Level V	Expert opinion without explicit critical appraisal, or based on physiology, bench research or ‘first principles’

RCT = randomised controlled trial

## Findings

**Figure 1:**
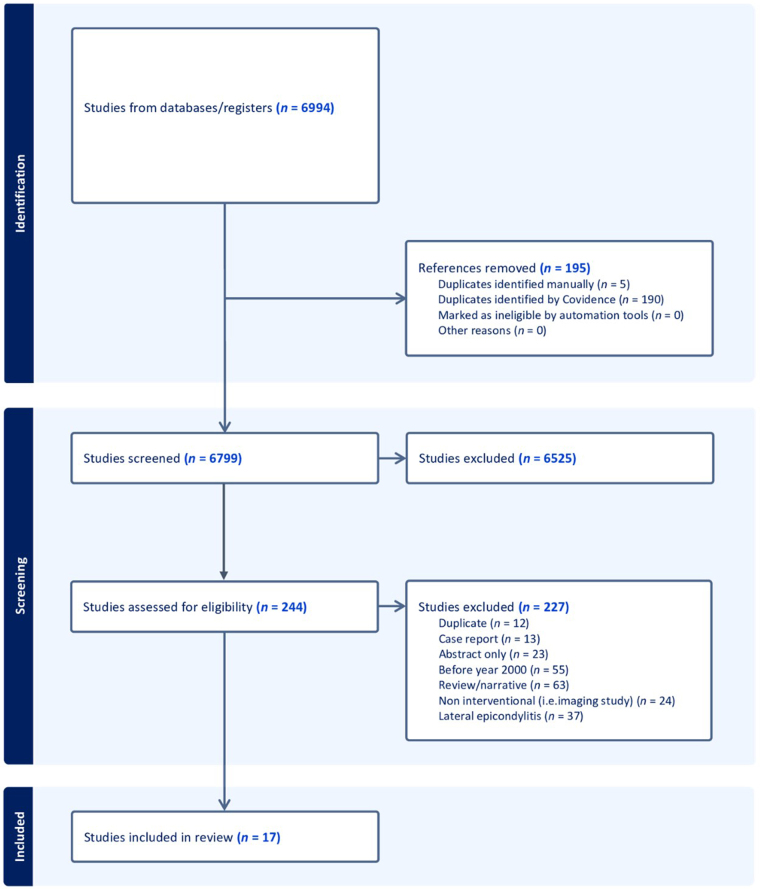
PRISMA flow chart for the included studies

Initially, 6,994 studies were identified. Of those, 195 duplicates were omitted leaving 6,799 studies for screening. The initial title and abstract screening excluded 6,555 studies with 244 studies left for full-text screening. Furthermore, the following 227 studies were excluded: duplicates (*n* = 12), case reports (*n* = 13), abstract-only studies (*n* = 23), studies before the year 2000 (*n* = 55), review/narrative articles (*n* = 63), noninterventional studies (e.g. imaging studies) (*n* = 24) and LE studies (*n* = 37). Seventeen surgical studies (442 patients, 475 elbows) met the inclusion criteria (Figure 1).

Table 3 enumerates the included surgical studies, the assessed techniques, patient demographics and individual level of evidence as reported by the relevant authors and as assessed by the MINORS assessment tool.^7–23^ Of the 17 studies included, 14 were retrospective, 2 were case–control and 1 was a prospective study. This distribution underscores the scarcity of high-quality RCTs in the existing literature, which limits the ability to draw robust conclusions. The median MINORS criteria for the included studies are 7 (range 3–14). The identified techniques ranged from open debridement to arthroscopic release and percutaneous techniques.

**Table 3:** The final included studies with different patient demographics and level of evidence

Study	Technique	Outcome measures	Follow-up duration (mean, range)	Patient demographics	Ulnar nerve affection	Duration before intervention (mean, range)	Level of evidence	MINORS/RoB criteria
Open techniques								
Shahid *et al*^7^ (2013, UK)	Open debridement without repair	Primary: VAS, DASH, MEPI and grip strength Secondary: RTW and complications rate	66 months (range 25–103)	Number: 15 patients (17 elbows) Mean age: 45 (range 23–65) Sex (male/female): 8 (53%)/7 (47%) Intervention on dominant hand: 15 (88%) Sports/occupation: not mentioned	Yes (*n* = 8, 47%)	Mean 10 months (range 8–20)	III-3 (retrospective)	5 (MINORS)
Kwon *et al*^8^ (2014, South Korea)	Open debridement (FETOR technique)	Primary: VAS, DASH and grip strength Secondary: complications rate	35.6 months (range 16–77)	Number: 20 (22 elbows) Mean age: 48 (range 29–58) Sex (male/female): 4 (20%)/16 (80%) Intervention on dominant hand: 14 (70%) Sports/occupation: not mentioned	Yes (*n* = 2, 9%)	Mean 34.2 months (range 8–240)	IV (retrospective)	8 (MINORS)
Schipper *et al*^9^ (2011, USA)	Open debridement (Nirschl technique) for country club elbows (combined tennis and golf elbows)	Primary: Nirschl score, numeric pain intensity scale and ASES Secondary: Nirschl grading system, patient satisfaction, return to sports and complications rate	11.7 years (range 5–19)	Number: 48 (53 elbows) Mean age: 45 (range 19–68) Sex (male/female): 26 (54%)/22 (46%) Intervention on dominant hand: 35 elbows (66%) Sports/occupation: 46 patients played sports (the most played sports were golf [*n* = 15], followed by tennis [*n* = 14] and weightlifting [*n* = 10])	Yes (*n* = 22, 41.5%)	>6 months	IV (retrospective)	8 (MINORS)
Han *et al*^10^ (2016, South Korea)	Open debridement	Primary: VAS, DASH, MEPI and grip strength Secondary: RTW, return to sports and complications rate	6.9 years (range 5–14)	Number: 55 (63 elbows) Mean age: 51 (range 34–77) Sex (male/female): 20 (35%)/36 (65%) Intervention on dominant hand: not mentioned Sports/occupation: not mentioned	No	Mean 24 months (range 12–120)	IV (retrospective)	7 (MINORS)
Gong *et al*^11^ (2010, South Korea)	Open Z-plasty lengthening of the FPO with submuscular ulnar nerve transposition	Primary: VAS, DASH and grip strength Secondary: Nirschl grading system, modified Wilson and Krout score and complications rate	38 months (range 24–48)	Number: 19 Mean age: 51 (range 29–77) Sex (male/female): 4 (21%)/15 (79%) Intervention on dominant hand: 7 (36%) Sports/occupation: preceding history of golf in seven, occupational overuse in one	Yes (*n* = 19, 100%)	>1 year	III-3 (retrospective)	7 (MINORS)
Erikson *et al*^12^ (2015, USA)	Open Z-plasty lengthening of the FPO	Primary: VAS, DASH Secondary: Nirschl grading system and satisfaction rates	14.8 months ± 4.7	Number: 12 (14 elbows) Mean age: 55 ± 9 Sex (male/female): 4 (33%)/8 (66%) Intervention on dominant hand: 11 (79%) Sports/occupation: 5 (3 for tennis and 2 for golf)	Yes (*n* = 2, 14.2%)	26.8 ± 14.8	IV (retrospective)	5 (MINORS)
Mooney *et al*^13^ (2019, USA)	Open debridement: Group A: ulnar nerve decompression Group B: ulnar nerve transposition	Primary: VAS, DASH, pinch and grip strength Secondary: ROM, two-point discrimination and complications rate	4.3 years (range 0.6–8.9)	Number: 14 (15 elbows). Group A: 3 patients (21%), Group B: 11 patients (79%) Mean age: 51 (range 31–57) Sex (male/female): 8 (57%)/6 (43%) Intervention on dominant hand: not mentioned Sports/occupation: not mentioned	Yes (*n* = 15, 100%)	–	III-3 (case–control)	9 (MINORS)
Grawe *et al*^14^ (2016, USA)	Open debridement with single suture anchor repair	Primary: VAS, quick DASH and OES Secondary: return to sports and complications rate	Median 40 months (range 12–67)	Number: 31 Mean age: 55 (range 29–65) Sex (male/female): 23 (74%)/8 (26%) Intervention on dominant hand: not mentioned Sports/occupation: not mentioned	Yes (*n* = 6, 19%)	>4 months	III-3 (retrospective)	7 (MINORS)
Vinod and Ross^15^ (2015, USA)	Open debridement with single suture anchor repair	Primary: MEPI Secondary: pain intensity scale and complications	1 year	Number: 60 Mean age: 52.5 Sex (male/female): 44 (73%)/16 (27%) Intervention on dominant hand: not mentioned Sports/occupation: Sports related in (76.7%), 33% played golf	Yes (*n* = 12, 20%)	144.2 weeks	III-3 (retrospective)	9 (MINROS)
Wu *et al*^16^ (2019, USA)	Open debridement with double-row anchor repair	Primary: VAS, MEPI and OES Secondary: time to pain-free state, time to FROM and Nirschl grading system	Mean clinical and telephone follow-up periods were 2.3 and 3.6 years. The minimum follow-up was 2 years	Number: 31 (33 elbows) Mean age: 46 (range 18–69) Sex (male/female): 20 (61%)/13 (39%) Intervention on dominant hand: not mentioned Sports/occupation: not mentioned	Yes (*n* = 15, 45%)	Mean 4.3 months (range 1.5–25.9)	IV (retrospective)	8 (MINORS)
Bohlen *et al*^17^ (2020, USA)	Group A: surgical debridement and subsequent anchor reattachment Group B: 2 leucocyte-rich PRB injections	Primary: VAS, MEPI, OES, time to pain-free status and time to FROM Secondary: Nirschl grading system	3.9 years	Number: 33. Group A: 18 (55%), Group B: 15 (45%) Mean age: Group A: 47.1 (range 16–62), Group B: 37.5 (range 16–64) Sex (male/female): 24 (72%)/9 (28%) Intervention on dominant hand: not mentioned Sports/occupation: not mentioned	No	Group A: mean 4.0 months (range 1.5–25.9) Group B: mean 6.3 months (range 1.8–45.0)	III-3 (retrospective)	14 (MINORS)
Arthroscopic techniques								
Oda *et al*^18^ (2018, Japan)	Arthroscopic release	Primary: VAS and DASH Secondary: Nirschl grading system and complications rate	16.2 months (range 6–28)	Number: 4 (5 elbows) Mean age: 48 (range 44–52) Sex (male/female): 2 (50%)/2 (50%) Intervention on dominant hand: 3 (60%) Sports/occupation: 2 office clerks, 1 furniture craftsman, and 1 car technician	No	>6 months	IV (retrospective)	3 (MINORS)
doNascimento *et al*^19^ (2017, Brazil)	Arthroscopic release	Primary: VAS, DASH and SF-36 Secondary: complications rate	17 months (range 6–48)	Number: 7 Mean age: 50 (range 36–67) Sex (male/female): 5 (71%)/2 (29%) Intervention on dominant hand: 6 (85%) Sports/occupation: not mentioned	No	Mean 2 years (range 8 months–4 years)	IV (retrospective)	3 (MINORS)
Kim *et al*^20^ (2023, South Korea)	Group A: open debridement without repair Group B: arthroscopic release	Primary: VAS, DASH and grip strength Secondary: operating time, range of motion, RTW and complications rate	20.2 months (range 12–58)	Number: 38 (44 elbows). Group A: 25 elbows (56%), Group B: 19 elbows (44%) Mean age: Group A 50 (range 29–72, Group B: 53 (range 27–70) Sex (male/female): 17 (38%)/27 (62%) Intervention on dominant hand: Group A: 17 (68%), Group B: 7 (36%) Sports/occupation: not mentioned	No	>6 months	III-3 (case–control)	13 (MINORS)
Percutaneous techniques								
Sahu *et al*^21^ (2017, India)	Percutaneous release	Primary: pain relief score and MEPI Secondary: patient satisfaction and complications rate	12 months	Number: 34 Mean age: 50 (range 30–60) Sex (male/female): 10 (29%)/24 (71%) Intervention on dominant hand: not mentioned Sports/occupation: not mentioned	–	>6 months	III-3 (prospective)	9 (MINORS)
Tasto *et al*^22^ (2016, USA)	Radiofrequency micro-tenotomy	Primary: VAS Secondary: complications rate	2.5 years (range 0.5–9)	Number: 11 Mean age: 50 Sex (male/female): not mentioned Intervention on dominant hand: not mentioned Sports/occupation: not mentioned	—	>6 months	III-3 (prospective)	12 (MINORS)
Lee *et al*^23^ (2022, South Korea)	Percutaneous embolisation (TAE technique)	Primary: VAS, and quick DASH Secondary: complications rate	Up to 6 months	Number: 10 (14 elbows) Mean age: 48 (range 29–58) Sex (male/female): 3 (30%)/7 (70%) Intervention on dominant hand: 8 (80%) Sports/occupation: not mentioned	—	Mean 31.3 months (range 4–86)	III-3 (retrospective)	6 (MINORS)

ADL  =  activities of daily life; ASES  =  American Shoulder and Elbow Surgeons score; DASH  =  Disabilities of the Arm, Shoulder and Hand; FETOR  =  fascial elevation and tendon origin resection; FPO  =  flexor pronator origin; FROM  =  full range of motion; MEPI  =  Mayo Elbow Performance Index; MINORS  =  Methodological Index For Non-randomised Trials; OES  =  Oxford Elbow Score; PRB  =  platelet-rich plasma; RoB  =  risk of bias; RTW  =  return to work; SF-36  =  Short Form 36; TAE  =  transcatheter arterial embolisation; VAS  =  visual analogue score

### Surgical techniques

#### Open surgical techniques

The open surgical debridement techniques are all predicated on the Nirschl technique for elbow tendinosis based on the identification and excision of pathologic tissue.^24^ The described open techniques include those involving FPO release only (with or without cortical drilling) as opposed to FPO release followed by anchor reattachment, and whether the surgical release was performed alongside a concurrent ulnar nerve procedure (decompression vs transposition).

#### Surgical debridement without bone drilling

Shahid *et al* retrospectively analysed outcomes following open surgical debridement in 17 elbows. The FPO was simply debrided without additional procedures.^7^ By contrast, Kwon *et al* described a technique modification with fascial elevation and tendon origin resection (FETOR) in their cohort of 22 elbows, 18% of whom had a concomitant LE.^8^ Both studies found significant improvements in pain and function following surgery. Shahid *et al* reported excellent outcomes including increased grip strength and high satisfaction postoperatively. Similarly, Kwon *et al* found major decreases in visual analogue score (VAS) pain scores and Disabilities of the Arm, Shoulder and Hand (DASH) disability scores. All patients but one were satisfied with their operation as per the criteria of the Nirschl and Pettrone elbow score.^8^

However, the immobilisation and rehabilitation protocols differed between the two studies. Whereas Shahid *et al* utilised no immobilisation and began aggressive physiotherapy immediately, Kwon *et al* applied a long arm splint for 2 days followed by a removable splint. Shahid *et al* reported an average return to work time of 8 weeks, whereas Kwon *et al* indicated that 90.9% of their cohort returned to their previous occupation. This comparison highlights a difference in the measure of outcome, with Shahid *et al* focusing on time to return and Kwon *et al* on the proportion of patients returning. Complication rates were also lower in Shahid *et al*’s study, with only one case of superficial wound infection compared with two cases of transient hypoesthesia in Kwon *et al*’s patients. Overall, both open debridement techniques resulted in excellent outcomes, but the addition of FETOR and concomitant procedures in Kwon *et al*’s study did not seem to confer extra benefit compared with simple debridement alone based on the parameters analysed in these two case series.

#### Surgical debridement with bone drilling

Both Schipper *et al* and Han *et al* reported on long-term outcomes for surgical treatment of ME, with some patients also having coexistent LE. Schipper *et al* demonstrated favourable outcomes even after a considerable mean follow-up of 11.7 years in their series of 53 country club elbows (combined tennis and golf elbow release).^9^ Similarly, Han *et al* presented a long-term follow-up cohort of 55 patients (63 elbows) with a mean follow-up of 6.9 years.^10^ The surgical techniques differed slightly, with Schipper *et al* utilising a single drill hole distal to the resected tissue, whereas specific details of Han *et al*’s technique were not provided. Immobilisation protocols also varied between the two studies.

In terms of outcomes, both studies demonstrated significant improvements in pain, function and grip strength scores. Schipper *et al* reported improvements in Nirschl tennis elbow score, American Shoulder and Elbow Surgeons (ASES) score, and VAS pain score, with 85% achieving good to excellent results. Han *et al* similarly found improvements in VAS pain, Mayo Elbow Performance Index (MEPI), DASH and grip strength. However, Han *et al* had a higher percentage of excellent to good Nirschl and Pettrone grades at 94% compared with Schipper *et al*’s 85%. Complication rates were low in both studies, with Schipper *et al* reporting 1.8% postoperative infection rate and Han *et al* noting 1.5% heterotopic ossification rate. Schipper *et al* did not report on return to work or exercise, whereas Han *et al* found a mean time of 2.8 months to return to work and 4.8 months to return to exercise.

#### Surgical debridement with ulnar nerve decompression/transposition

Gong *et al* and Erikson *et al* both described techniques involving Z-plasty lengthening of the FPO combined with ulnar nerve procedures for concomitant cubital tunnel syndrome. Gong *et al* performed submuscular ulnar nerve transposition on all 19 patients in their study with a mean follow-up of 38 months.^11^ Erikson *et al*, however, only transposed the nerve sub-muscularly in 2 of 12 patients (14 elbows) in their series with a mean 14.8-month follow-up.^12^ The postoperative rehabilitation protocols differed, with Gong *et al* using a sling for 2 weeks and allowing return to full activity at 3 months, whereas Erikson *et al* utilised a counterforce brace for 6 months. Complication rates were low, with Gong *et al* reporting 10.5% mild numbness and Erikson *et al* having no complications. In terms of outcomes, both studies reported excellent to good results based on Nirschl and Pettrone grading.

Mooney *et al* compared ulnar nerve decompression vs transposition specifically, finding generally superior outcomes for the transposition group in DASH, VAS, strength and range of motion (ROM) measures, although the difference was not statistically significant for all parameters.^13^ Their study had a longer mean follow-up of 4.3 years. Postoperative splinting was not routinely used, and 13% of transposition patients developed haematomas requiring treatment. In summary, although the Z-plasty lengthening techniques were similar between Gong *et al* and Erikson *et al*, differences were reported in ulnar nerve management, rehabilitation protocols and outcome measures. Mooney *et al*’s study directly compared decompression and transposition, favouring transposition despite a higher complication rate.

#### Surgical debridement with subsequent reattachment

Grawe *et al*, Vinod and Ross, and Wu *et al* all described techniques involving suture anchor repair/reattachment for ME. Grawe *et al* utilised one or two double-loaded anchors in 31 patients with 6–8 weeks immobilisation.^14^ Vinod and Ross performed single anchor repair on 60 patients with accelerated rehabilitation without immobilisation.^15^ Wu *et al* employed a double-row 1.9mm anchor repair on 33 elbows after routine arthroscopy, immobilising for 1 week in a slab then 6 weeks in braces.^16^ In terms of outcomes, Grawe *et al* reported median QuickDASH of 45, Oxford Elbow Score (OES) of 2.3, satisfaction 1/10, pain 1/10, with 86% return to sport at 4.5 months. Vinod and Ross found significant MEPI and pain intensity improvement, with one revision case. Wu *et al* had mean VAS improvement of 4.9, MEPI of 95.1, OES of 45.3, 87.5 days to pain free, 114.1 days to full range of motion (FROM), 94% excellent to good Nirschl scores and 97% back to premorbid activity.

Bohlen *et al* compared anchor repair with PRP injection, with the surgical group allowing immediate mobilisation like the PRP group.^17^ Striking differences were faster time to pain free (56.2 vs 108 days) and FROM (42.3 vs 96.1 days) favouring PRP, although trends for better VAS/MEPI/OES in the PRP group did not reach significance. Ultimately, 94% of the surgical group achieved successful Nirschl outcome vs 80% of the PRP group. Although all studies showed generally good outcomes with suture anchor repair techniques, variations in anchor configuration, rehabilitation protocols and precise outcome measures were reported. Bohlen *et al*’s comparison suggests PRP may provide faster recovery of function/pain, although slightly lower rate of successful Nirschl outcomes compared with surgical repair.

### Arthroscopic techniques

Oda *et al* and doNascimento *et al* reported on small case series of arthroscopic release for ME involving five elbows and seven patients, respectively. Oda *et al* did not utilise any postoperative immobilisation, whereas doNascimento *et al* had patients use a sling for 3–5 days.^18,19^ Return to full activity was allowed at 6–8 weeks for Oda *et al* and 10–12 weeks for doNascimento *et al*. Complication rates were low, with one case of transient medial antebrachial cutaneous nerve (MACN) irritation for Oda *et al* and one major haematoma for doNascimento *et al*. Both studies demonstrated significant improvements in VAS pain and DASH scores postoperatively. Oda *et al* additionally reported excellent or good Nirschl outcomes in all cases.

Kim *et al* compared a larger cohort of 44 elbows undergoing either open (56%) or arthroscopic (44%) debridement, with no immobilisation but 6 weeks of restricted activity for both groups.^20^ There were no significant differences between the open and arthroscopic groups in terms of DASH, VAS or grip strength improvement. Excellent to good outcomes were achieved in 80% of open cases and 84% of arthroscopic cases. Although the return to work time trended faster for arthroscopic cases (9.6 vs 11.1 weeks), this difference did not reach statistical significance. In summary, all three studies showed the efficacy of arthroscopic debridement for ME, with outcomes comparable with open techniques based on the data provided. Rehabilitation protocols varied, although most allowed relatively early return to activity.

### Percutaneous techniques

Sahu *et al*, Tasto *et al* and Lee *et al* described minimally invasive percutaneous or probe-based treatment techniques for ME. Sahu *et al* performed a percutaneous release on 34 patients via a 0.5cm incision, completely dividing the FPO without bone removal or debridement.^21^ Tasto *et al* utilised radiofrequency micro-tenotomy in 11 patients, creating a grid-like pattern of perforations in the tendon.^22^ Lee *et al* described an ultrasound-guided percutaneous embolisation technique in 14 elbows, injecting an embolic agent into specific arteries supplying the area.^23^ In terms of post-procedure protocols, none of the studies utilised immobilisation. Sahu *et al* and Tasto *et al* allowed immediate ROM, with return to full activities by 8 weeks and 6–9 weeks, respectively. Lee *et al* did not specify a rehabilitation protocol.

Regarding outcomes, Sahu *et al* reported 88.2% full satisfaction and 11.7% partial satisfaction at 12 months, with all patients returning to premorbid levels by 8 weeks on average. Tasto *et al* found significant VAS improvement from 6.1 to 1.3 at mean 2.5-year follow-up, with durable pain relief up to 9 years. Lee *et al* achieved 85.7% clinical success, with significant 6-month improvements in VAS and QuickDASH sustained in 90% at 12 months. Complication rates were low across all three studies. Sahu *et al* and Tasto *et al* reported no complications, whereas Lee *et al* noted no elbow-related complications from the percutaneous embolisation procedure. In summary, these minimally invasive techniques provided good outcomes for ME while allowing early ROM and reasonably rapid return to activities, although follow-up duration varied between studies.

### Primary outcomes

The most utilised primary outcome measures included VAS in 15 studies, DASH in 9 studies and MEPI in 7 studies, which were all significantly improved after their relevant procedures (*p *> 0.05). Other less commonly used primary outcomes measures included grip and/or pinch strength in seven studies, OES in three studies, ASES in one study, Short Form 36 (SF-36) in one study and Nirschl score in one study. The weighted means were calculated for the three most used primary outcome measures (VAS, DASH and MEPI). The results showed a universal improvement in outcome measures after surgical interventions whether open or arthroscopic or percutaneous. The weighted preoperative VAS was 5.99 ± 0.55 (range 2.5–8.3), whereas weighted postoperative VAS was 1.65 ± 0.64 (range 0.3–2.8) (*p* < 0.01). The weighted preoperative DASH was 38.26 ± 3.06 (range 1.2–59.1), whereas the postoperative DASH was 10.24 ± 2.79 (range 6.2–23) (*p* < 0.01). The weighted preoperative MEPI was 43.34 ± 12.47 (range 31.9–72) and postoperative weighted MEPI was 91.44 ± 1.83 (range 83.9–97) (*p* < 0.01). A summary of pre- and postoperative primary functional outcomes is outlined in Table 4.

**Table 4:** Comparison between preoperative and postoperative primary outcome measures for each study with levels of significance if mentioned

Study	Preoperative primary measures	Postoperative primary measures	Significance
Shahid *et al*^7^	VAS: —	VAS: 9 (range 0–50)	—
	DASH: 42.6 (range 24–74)	DASH: 12.9 (range 0–36)	*p* < **0.001**
	MEPI: —	MEPI: 97 (range 65–100)	—
	Grip strength: 27 ± 11kg	Grip strength: 27 ± 11kg	*p* < **0.001**
Kwon *et al*^8^	VAS (average): 6.8 ± 1.7	VAS (average): 0.5 ± 1.0	*p* < **0.01**
	VAS at rest: 3.4 ± 3.0	VAS at rest: 0.2 ± 0.5	*p* < **0.01**
	VAS during activity: 8.0 ± 1.6	VAS during activity: 1.4 ± 1.5	*p* < **0.01**
	DASH: 51.6 ± 18.0	DASH: 8.0 ± 11.1	*p* < **0.01**
	Grip strength: 53.7 ± 30.3% of the uninvolved arm	Grip strength: 97.3 ± 19.8% of the uninvolved arm	*p* < **0.01**
Schipper *et al*^9^	Nirschl score: 16.7 ± 2.3	Nirschl score: 70.8 ± 1.8	*p* < **0.01**
	VAS: 8.8 ± 0.2	VAS 1.7 ± 0.3	*p* < **0.01**
	ASES: 45.2 ± 2.3	ASES: 90.4 ± 2.1	*p* < **0.01**
Han *et al*^10^	VAS: 8.5	VAS: 2.4	*p* < **0.01**
	DASH: 57	DASH: 23	*p* < **0.01**
	MEPI: 72	MEPI: 88	*p* < **0.01**
	Grip strength: 30lb	Grip strength: 43lb	*p* < **0.01**
Gong *et al*^11^	VAS at rest: 3.7 (range 2–5)	VAS at rest: 0.3 (range 0–2)	*p* < **0.001**
	VAS during activity: 6.6 (range 4–9)	VAS during activity: 2.1 (range 1–4)	*p* < **0.001**
	DASH: 42.2 (range 27–59)	DASH: 23.5 (range 12–49)	*p* < **0.001**
	Grip strength: 18.6kg (range 9.1–37.3)	Grip strength: 22.8kg (range 12.7–47.3)	*p* < **0.001**
Erikson *et al*^12^	VAS at rest: —	VAS at rest: 0.78 ± 1.5	—
	VAS during activity: —	VAS during activity: 1.71 ± 1.72	
	DASH: —	DASH: 37.73 ± 13.11	
Mooney *et al*^13^	VAS: 2.5	VAS: 8.0 (decompression), 1.2 (transposition) (*p* = 0.002)	—
	DASH: 24.1	DASH: 68.2 (decompression), 13.1 (transposition) (*p* = 0.004)	—
	Key pinch strength: —	Key pinch strength: 4.9 kg (decompression), 8.3kg (transposition) (*p* = 0.160)	—
	Grip strength: —	Grip strength: 19.8kg (decompression), 37.4kg (transposition) (*p* = 0.131)	—
Grawe *et al*^14^	VAS: —	VAS: Median 1 (range 1–4)	—
	Quick DASH: —	Quick DASH: Median 2.3 (range 0–38.6)	—
	OES: —	OES: Median 45 (range 22–48)	—
Vinod and Ross^15^	MEPI: 58 ± 7.7	MEPI: 88 ± 7.8	*p* < **0.001**
	Pain scale: 2.2 ± 0.3	Pain scale: 0.6 ± 0.5	
Wu *et al*^16^	VAS: 5.8	VAS: 0.9	*p* < **0.01**
	MEPI: —	MEPI: 95.1 (range 65–100)	—
	OES: —	OES: 45.3 (range 25–48)	—
Bohlen *et al*^17^	VAS: —	VAS: 4.7 (surgery) vs 3.7 (PRP)	*p* = 0.12
	Time to pain-free status: —	Time to pain-free status: 108.0 days (surgery) vs 56.2 days (PRP)	*p* < **0.01**
	Time to FROM: —	Time to FROM: 96.1 days (surgery) vs 42.3 days (PRP)	*p* < **0.01**
	MEPI: —	MEPI: 93.5 (surgery) vs 92.3 (PRP)	*p* = 0.30
	OES: —	OES: 42.2 (surgery) vs 45.9 (PRP)	*p* = 0.18
Oda *et al*^18^	VAS at rest: 5.8 ± 4.4	VAS at rest: 0.3 ± 0.6	—
	VAS during activity: 8.3 ± 1.9	VAS during activity: 2.3 ± 2.2	—
	DASH: 59.1 ± 24.3	DASH: 7.5 ± 10.3	—
doNascimento *et al*^19^	VAS at rest: 7.8 ± 4.4 (range 3–10)	VAS at rest: 1.9 ± 2.3 (range 0–6)	*p* = **0.006**
	DASH: 38.3 ± 16.8 (range 18.7–63.3)	DASH: 6.3 ± 1.3 (range 5–8)	*p* = **0.04**
	SF-36: 67.7 ± 27.6	SF-36: 78.2 ± 22.4	—
Kim *et al*^20^	VAS: 8.5 ± 1.5 (open), 8.2 ± 1.3 (arthroscopic) (*p* = 0.25)	VAS: 1.0 ± 0.8 (open), 1.1 ± 0.6 (arthroscopic) (*p* = 0.47)	—
	DASH: 44.8 ± 15.8 (open), 43.9 ± 18.9 (arthroscopic) (*p* = 0.84)	DASH: 12.5 ± 12.3 (open), 13.2 ± 10.6 (arthroscopic) (*p* = 0.75)	—
	Grip strength: 72.2 ± 13.1% (open), 66.8 ± 20.4% (arthroscopic) to the uninvolved side (*p* = 0.41)	Grip strength: 84.4 ± 5.6% (open), 83.6 ± 7.4% (arthroscopic) to the uninvolved side (*p* = 0.59)	—
Sahu *et al*^21^	Pain relief: Not applicable	Pain relief: achieved on average 8 weeks	—
	MEPI score: —	MEPI score: Excellent in 88.23% and good in 11.76%	—
Tasto *et al*^22^	VAS: 6.1	VAS: 1.3	*p* < **0.01**
Lee *et al*^23^	VAS: 7.6 ± 1.2	VAS (12 months): 0.9 ± 0.8	*p* < **0.01**
	Quick DASH: 71.9 ± 14.4	Quick DASH: 8.8 ± 10.7	*p* < **0.01**

ASES  =  American Shoulder and Elbow Surgeons score; DASH  =  Disabilities of the Arm, Shoulder and Hand; FROM  =  full range of motion; MEPI  =  Mayo Elbow Performance Index; OES  =  Oxford Elbow Score; SF-36  =  Short Form 36; VAS  =  visual analogue score.

### Complications

Total complication rate was 3.1% (*n* = 14 of 442, range 1.5–20%) with 1% (*n* = 4) MACN neuropraxia that spontaneously resolved, 1% (*n* = 4) haematomas that required open washout and drainage, 0.2% (*n* = 1) superficial infection treated with antibiotics, 0.2% (*n* = 1) retear after repair with revision surgery, 0.2% (*n* = 1) heterotopic ossification that did not lead to further functional sequalae and 0.8% (*n* = 3) patients with radial artery puncture site pain. Excluding those with radial artery puncture site pain in Lee *et al* study, the elbow-related complications rate is 2.4% (*n* = 11 of 442).^23^ The lowest elbow-related complication rates were reported with percutaneous techniques with no complications (0%) of 55 cases. The highest elbow-related complication rates were reported with ulnar nerve transposition in 11.1% (*n* = 4 of 36), and in arthroscopic release with 6.4% (*n* = 2 of 31).

### Impact of preoperative injections

Four studies investigated whether there is a correlation between preoperative injections and functional outcomes postoperatively.^9–11,14^ Gong *et al* reported that the patients received a mean number of 3.5 (range 1–10) steroid injections before referral.^11^ This was the only study that reported that there was a correlation between the number of preoperative injections and postoperative satisfaction, which was significant with a Spearman’s coefficient of 0.564 (*p* = 0.012), indicating that those who had received more injections had a better postoperative result.

On the contrary, Han *et al* reported that their cohort had received a mean of 5 injections (range 3–10) before surgery and their analysis showed that the number of injections before the surgery did not correlate with the improvement of the mean VAS, MEPI, DASH scores and the mean grip strength of the affected side.^10^ Schipper *et al* mentioned that steroid injections were administered to 64% of the elbows (*n* = 34 of 53) with a mean of 2 injections (range 1–8).^9^ Likewise, they reported that there was no significant difference in postoperative Nirschl tennis elbow, ASES or numeric pain intensity scale scores between patients who received fewer or more than three injections. These findings were further corroborated by Grawe *et al* who reported that 45% (*n* = 14 of 31) of their patients had at least one steroid injection and that 23% (*n* = 7) had a preoperative PRP injection, but demonstrated a history of previous injection did not impact postoperative results. The evidence suggests injections do not provide lasting benefit and cannot make the underlying condition better in preparation for surgery.

### Impact of ulnar neuritis

Only two studies investigated the correlation between a pre-existent ulnar neuritis and post-procedure outcomes.^14,16^ Grawe *et al* series included 19% (*n* = 6) who had clinical signs and symptoms consistent with ulnar neuritis preoperatively.^14^ They found that ulnar nerve symptoms did not show a statistically significant association with any measured outcome variable (*p *> 0.05). Wu *et al* included 45% (*n* = 15 of 33) of patients with pre-existing ulnar neuritis diagnosis.^16^ Of those, 46% (*n* = 7 of 15) were classified as type IIA and 54% (*n* = 8) as type IIB depending on whether they had subjective and/or objective ulnar neuritis symptoms as per Gabel and Morrey criteria.^1^ Similarly, they showed no difference in outcomes between those with and without ulnar neuritis preoperatively (*p* = 0.67).

## Discussion

This systematic review comprehensively identified 17 studies, comprising 442 patients and 475 elbows, on the surgical management of ME from 2000 to date. Median nonoperative treatment duration was 6 months (range 1.5–12). When surgery was contemplated for those refractory cases, there was a universal improvement in all functional outcomes with a low overall complications rate of 3.1%. Both delayed and accelerated rehabilitation protocols led to high satisfaction rates, but no superiority comparative studies were identified for this regard in our review. No study found a correlation between preoperative concomitant ulnar neuritis and postoperative functional outcomes, and only one of four studies found a correlation between number of preoperative injections and postoperative outcomes.

To the best of our knowledge, only one systematic review interrogated the available literature on different surgical techniques for ME.^25^ In total, 16 studies were identified during the review period from 1980 to 2020. However, it is important to note that five studies included in that review were conducted before 2000, which marks the beginning of our review period. In addition, we identified four more studies during the same review period (2000–2020) and two more studies published after the conclusion of their review period in 2020. This systematic review by Arevalo *et al* identified 479 elbows with a complication rate of 4.3%. The review’s main objectives were to assess success rates for return to sports and work, which were 81–100% and 66.7–100% respectively. The correlation between preoperative injections and concomitant ulnar neuritis on postoperative functional outcomes was the focus of our review.

In the pooled 17 studies, surgery was almost always preceded by a trial of injections; however, it could be first line for selective cases. For example, Grawe *et al* refrained from giving any injections to those who had associated ulnar nerve symptoms because it was deemed unlikely to be effective in this setting.^14^ Similarly, Bohlen *et al*, in their comparative study of PRP vs open surgical debridement of ME, concluded that, based on PRP offering a comparable final outcome and shorter recovery time, it is a reasonable treatment option for ME without concomitant ulnar neuritis.^17^ Although surgery had a higher success rate in their series (94% vs 80%), they recommended that it should be reserved for recalcitrant cases (given the significantly longer recovery time required as compared with injections).

In terms of the identified techniques, functional outcomes improved significantly regardless of cortical drilling, reattachment of FPO and whether or not an ulnar nerve procedure was performed. In terms of other prognostic predictors, Kwon *et al* found that outcomes measures such as the VAS, DASH and grip strength were consistently better whenever FPO calcifications were noted intraoperatively.^8^ They postulated that one possible explanation for this is that calcified tissue is more easily identifiable and therefore results in a more thorough excision, thus leading to better outcomes. Only one study investigated the effect of age on surgical outcomes, and it was found that older age at the time of surgery was a significant predictor of better functional outcomes.^14^

As regards to the routine reattachment of the FPO, the rationale behind this is predicated on enhancing the healing process as well as restoring elbow stability, which is essential for individuals who engage in activities that require a strong and stable forearm, such as swinging a golf club. Grawe *et al* showed a prominent level of satisfaction and good pain relief with this operative intervention; however, with a protracted recovery and return to baseline sports activities that takes up to 4.5 months.^14^ Moreover, two of four identified anchor reattachment studies applied an elbow brace or a sling up to 6 weeks to protect the repair.^14,16^

In terms of pre-existing ulnar neuritis, nine studies identified in this systematic review included patients with preoperative cubital tunnel syndrome. The diagnosis was either through clinical examination or through electrophysiological studies. It is well established that even with objective signs, nerve conduction studies (NCS) might still be negative.^26^ Gong *et al* included 19 patients in their surgical cohort of myofascial release and concomitant ulnar nerve transposition.^11^ Of those, 36% (*n* = 7) were grade I (mild parathesia and no weakness) and 64% (*n* = 12) were grade IIa (moderate parathesia and mild weakness) according to the McGowan classification.^27^ It is worthwhile mentioning that only 50% of grade IIa patients (*n* = 6 of 12) were confirmed on NCS. Similarly, Shahid *et al* reported that 47% (*n* = 8 of 17) of their patients had symptoms of ulnar neuropathy with hypoesthesia in the distribution of the ulnar nerve, with seven having a positive Tinel’s sign.^7^ All eight patients had NCS, which failed to demonstrate ulnar nerve dysfunction. Based on this, we cannot recommend a routine preoperative nerve conduction or electrophysiological study to investigate for ulnar neuritis.

Two studies were identified in this systematic review that assessed the effect of pre-existing ulnar neuritis on postoperative functional outcomes, with none of them finding a statistically significant correlation.^14,16^ However, earlier studies had a conflicting report of worse outcomes with coexistent ulnar neuritis. Gabel and Morrey reported worse outcomes in those with pre-existing severe ulnar neuritis after surgical debridement and ulnar nerve decompression.^1^ Similarly, Kurvers and Verhaar retrospectively reviewed 40 patients and noted favourable outcomes only in the 25 elbows that did not have coexistent ulnar neuritis.^28^

The decision of a concomitant ulnar nerve procedure (whether decompression or transposition) varied among different authors, with some advocating routine ulnar nerve decompression/transposition whenever there are pre-existing symptoms, with the decision to perform *in situ* decompression or transposition based on intraoperative stability of the ulnar nerve.^13^ Mooney *et al* showed, in the context of ME, the outcome measures were generally superior for the transposition group as opposed to the decompression group. Contrarily, Wu *et al* showed that there was no difference in outcomes between those who had decompression and those who had a transposition (*p* = 0.49) in a small series of ten patients.^16^ Other authors recommended that an ulnar nerve procedure should be made based solely on the intraoperative presence of focal compression and whether the ulnar nerve is sublaxing or not regardless of preoperative existing symptoms.^9,15^ In Shahid *et al*’s series, none of the patients who had a preoperative clinical diagnosis of concomitant cubital tunnel had an ulnar nerve decompression or transposition as no focal compression or subluxation was noted intraoperatively.^7^ In all patients, these symptoms resolved following surgery. It is also worthwhile noting that this systematic review highlights that a relatively higher complications rate was encountered with procedures involving a concomitant ulnar nerve procedure (11.1%).

Arthroscopic treatment was postulated to offer reduced surgical morbidity and the ability to assess for concomitant intra-articular pathology. Only one author advocated routine elbow arthroscopy to be performed for all cases to rule out medial ulnar collateral ligament or other intra-articular pathology before the open procedure. However, it was not mentioned whether that changed the surgical plan in their study.^16^ A comparative study by Kim *et al* found similar excellent outcomes for either the open technique or the arthroscopic release, with a trend for quicker return to work in the arthroscopy group that was not statistically significant (*p* = 0.068).^20^ One caveat of arthroscopic procedure is of course the learning curve and the inability to address a concomitant ulnar nerve pathology without converting to an open procedure.

Percutaneous procedures have been described to reduce morbidity with the surgical procedures. We have identified three percutaneous surgical techniques with 0% elbow-related complications reported.^21–23^ The procedures were performed under local anaesthesia and patients were allowed to return to work and sports at once after the procedure. That being said, Cho *et al* described a percutaneous release for both ME and LE patients.^29^ Their series included 41 ME or LE patients and despite reporting an excellent to good outcome in 100% of the patients, a 7% complication rate (*n* = 3 of 41), with subcutaneous seroma in 2 patients that was managed by suction drainage and persistent pain in 1 patient, was reported. Nonetheless, this study was omitted from our systematic review outcomes due to a lack of stratification by the authors concerning ME and LE subgroups.

The predominance of retrospective studies in this review as well as the low cohort sizes highlights a significant limitation in the current evidence base for surgical management of ME. The absence of RCTs reduces the reliability of the findings and emphasises the need for more rigorous research designs in future studies.

## Conclusions

The objective of this systematic review was to provide an evidence-based overview of contemporary surgical techniques for ME. Our findings indicate that all identified techniques, whether open, arthroscopic or percutaneous approaches, prove favourable surgical outcomes and low complication rates. Notably, percutaneous techniques showed the quickest recovery and least complications. Selective ulnar nerve decompression or transposition is conducted based on intraoperative findings, when necessary, as routine ulnar nerve procedures could not be recommended based on the studies reviewed.

Despite the comprehensive search strategy employed, the quality of the included studies is still a limitation. All 17 studies identified in this review were of low to moderate quality (at best, level III evidence) and lacked RCTs.

Consequently, we advocate for a future high-quality comparative study to offer more definitive guidance for managing this condition. Specifically, a multicentric RCT could address whether percutaneous techniques lead to superior functional outcomes compared with open or arthroscopic methods, with a focus on recovery time and complication rates. Given the low prevalence of ME, a multicentric study will be well suited for this limitation to enable a well-powered study.
